# Deficient Regulatory T Cell Activity and Low Frequency of IL-17-Producing T Cells Correlate with the Extent of Cardiomyopathy in Human Chagas' Disease

**DOI:** 10.1371/journal.pntd.0001630

**Published:** 2012-04-24

**Authors:** Paulo Marcos Matta Guedes, Fredy Roberto Salazar Gutierrez, Grace Kelly Silva, Renata Dellalibera-Joviliano, Gerson Jhonatan Rodrigues, Lusiane Maria Bendhack, Anis Rassi, Anis Rassi, André Schmidt, Benedito Carlos Maciel, José Antonio Marin Neto, João Santana Silva

**Affiliations:** 1 Department of Microbiology and Parasitology, Federal University of Rio Grande do Norte, Natal, Brazil; 2 Department of Biochemistry and Immunology, School of Medicine of Ribeirão Preto, University of São Paulo, São Paulo, Brazil; 3 School of Medicine, University Antonio Narino, Bogota, Colombia; 4 Integrated Faculty Fafibe and Department of Surgery and Anatomy, School of Medicine of Ribeirão Preto, University of São Paulo, São Paulo, Brazil; 5 Department of Pharmacology, School of Medicine of Ribeirão Preto, University of São Paulo, São Paulo, Brazil; 6 Department of Physical and Chemistry, School of Pharmaceutical Sciences of Ribeirão Preto, University of São Paulo, São Paulo, Brazil; 7 Division of Cardiology, Anis Rassi Hospital, Goiânia, Brazil; 8 Division of Cardiology, School of Medicine of Ribeirão Preto, University of São Paulo, São Paulo, Brazil; University of Massachusetts Medical School, United States of America

## Abstract

**Background:**

Myocardium damage during Chagas' disease results from the immunological imbalance between pro- and production of anti-inflammatory cytokines and has been explained based on the Th1–Th2 dichotomy and regulatory T cell activity. Recently, we demonstrated that IL-17 produced during experimental *T. cruzi* infection regulates Th1 cells differentiation and parasite induced myocarditis. Here, we investigated the role of IL-17 and regulatory T cell during human Chagas' disease.

**Methodology/Principal Findings:**

First, we observed CD4^+^IL-17^+^ T cells in culture of peripheral blood mononuclear cells (PBMC) from Chagas' disease patients and we evaluated Th1, Th2, Th17 cytokine profile production in the PBMC cells from Chagas' disease patients (cardiomyopathy-free, and with mild, moderate or severe cardiomyopathy) cultured with *T. cruzi* antigen. Cultures of PBMC from patients with moderate and severe cardiomyopathy produced high levels of TNF-α, IFN-γ and low levels of IL-10, when compared to mild cardiomyopathy or cardiomyopathy-free patients. Flow cytometry analysis showed higher CD4^+^IL-17^+^ cells in PBMC cultured from patients without or with mild cardiomyopathy, in comparison to patients with moderate or severe cardiomyopathy. We then analyzed the presence and function of regulatory T cells in all patients. All groups of Chagas' disease patients presented the same frequency of CD4^+^CD25^+^ regulatory T cells. However, CD4^+^CD25^+^ T cells from patients with mild cardiomyopathy or cardiomyopathy-free showed higher suppressive activity than those with moderate and severe cardiomyopathy. IFN-γ levels during chronic Chagas' disease are inversely correlated to the LVEF (P = 0.007, r = −0.614), while regulatory T cell activity is directly correlated with LVEF (P = 0.022, r = 0.500).

**Conclusion/Significance:**

These results indicate that reduced production of the cytokines IL-10 and IL-17 in association with high levels of IFN-γ and TNF-α is correlated with the severity of the Chagas' disease cardiomyopathy, and the immunological imbalance observed may be causally related with deficient suppressor activity of regulatory T cells that controls myocardial inflammation.

## Author Summary

Dilated cardiomyopathy is one of the clinical forms of Chagas' disease (CD) after the infection caused by the parasite *Trypanosoma cruzi*. Even though strategies adopted in most Latin-American countries in the last decades towards vector control have been effective in reducing the incidence of CD, active transmission is maintained in some regions, and secondary prevention approaches are still required for the infected patients, mostly because the specific anti-parasitic medications are toxic and perhaps of limited efficacy in chronically infected individuals. Moreover, there are no markers to predict the risk of developing dilated cardiomyopathy in asymptomatic, chronically infected patients, although the failure in the mechanisms that control the immune response can be involved in the development of Chagas' heart disease. In this study we show that preserved activity of regulatory T cells and the production of the cytokine IL-17 are connected with a more benign evolution of the disease, which brings a new understanding on the mechanisms associated with progression of CD.

## Introduction

At the present time, about 7.7 million people are infected and 28 million are at risk of being infected with *Trypanosoma cruzi* in Central and South America [1]–[3]. This hemoflagellate protozoan is the etiological agent of Chagas' disease. Most of the infected individuals remain asymptomatic during chronic infection (60–70%), characterizing the indeterminate form of the disease. Conversely, 30–40% of chronically infected patients progress to cardiac and/or digestive pathologic involvement [4], [5], and prognostic markers for heart disease progression are required.

A balanced immune response during *T. cruzi* infection is critical to control the parasite burden in heart and digestive tissues [6], [7]. Production of pro-inflammatory cytokines is required for activation of the effector T lymphocytes responses and is associated with the pathogenesis of Chagas' disease cardiomyopathy (CC), while regulatory cytokines (mainly IL-10) are related to protection [8], [9]. Peripheral blood mononuclear cells (PBMC) from patients with CC produce more IFN-γ, TNF-α and IL-6, and less IL-4 and IL-10, compared to individuals with the indeterminate form of the disease [1], [3], [7], [10]–[14]. However, other studies failed to demonstrate any correlation between production of Th1 and Th2 cytokines profile and the clinical stages of Chagas' disease [15], being that further investigations to elucidate such mechanisms are necessary, one aim of this work.

Regulatory T cells (Treg) are an important source of regulatory cytokines and are involved in the control of the local inflammatory response and in avoiding extensive tissue destruction. However, their presence in the site of infections is frequently regarded as an inducer of parasite persistence [16]. Treg are able to migrate to the site of cardiac inflammation triggered by *T. cruzi*, and to suppress the effector function of CD4 and CD8 T cells during infectious processes [17]. They suppress the proliferation of effector T cells (CD4^+^CD25^−^) when co-cultured, and can also inhibit the activation of auto-reactive T cells through the expression of co-inhibitory molecules (CTLA-4) and the production of suppressive cytokines (IL-10, TGF-β, IL-35) [12], [18], [19]. Recent studies suggest that indeterminate Chagas' disease patients have higher frequency of CD4^+^CD25^high^ T cells in comparison to cardiac and non-infected individuals in their peripheral blood [20], [21]. Consequently, the measurement of CD4^+^CD25^high^ T cells suppressive activity in patients with indeterminate and cardiac forms of disease could be an important tool to evaluate a regulatory mechanism that prevents cardiac damage, which was another aim of this work. Treg do not seem to play a major role in regulating the effector responses of CD8 T cells in the myocardium during the acute and chronic experimental *T. cruzi* infection, since the blockade of CD25 did not change the inflammatory response or parasite burden in mice [13], [22], [23]. However, the treatment with anti-GITR resulted in increased mortality, TNF-α production, and myocarditis with enhanced migration of CD4, CD8, and CCR5 leukocytes to the heart in the *T. cruzi* infected mice [13]. If Treg could be involved in the control of immune response and cardiac disease progression in Chagas' disease patients is other aim of this work.

An additional lineage of effector CD4^+^ T helper lymphocytes, with potential regulatory properties, produces IL-17A that acts in several cells types leading the production of GM-CSF, IL-1, IL-6, and TNF-α, activation of NOS2, metalloproteinases and chemokines, resulting in leukocytes recruitment [24]–[27]. Treatment of *T. cruzi* infected mice with anti-IL-17A mAb lead to increased myocarditis, premature mortality, and decreased parasite load in the heart, suggesting that IL-17 controls the host resistance. Also, IL-17 regulates Th1 cells differentiation, cytokine and chemokine production and the influx of inflammatory cells to the heart tissue [28]. IL-17A^−/−^ mice infected with *T. cruzi* had a lower survival rate, multiple organ failure, and sustained parasitemia compared with wild-type mice, indicating that IL-17A is crucial to leukocyte activation that are critical for parasite killing [29]. Although it is not very clear, it seems to be a relationship between Tregs and Th17 cells. Differentiation of Th17 in the presence of Treg leads to increased specific cytokine release, what could be due the consumption of IL-2 [30], [31]. Similarly, Treg cell depletion results in a reduced frequency of IL-17 producers through modulation of IL-2 [32]. In addition, Treg can also be converted into a variety of T effector cells, including Th17 cells [33].

The purpose of the present study was to analyze the potential participation of IL-17 and Treg in the development of different clinical manifestations of human chronic Chagas' heart disease. Our hypothesis was that patients with chronic Chagas' disease undergoing cardiomyopathy produce increased levels of IL-17 and have a reduced frequency or suppressive activity of Treg compared with those patients with the indeterminate form of the disease. We provide novel information about immunological mechanisms involved in the human *T. cruzi* infection that could be used for the development of chemotherapies, as well as for the evaluation of prognostic markers of disease.

## Methods

### Patients

The inclusion of the 39 subjects (10 controls) in our investigation had the prior approval of an institutional ethics committee (Hospital das Clínicas de Ribeirão Preto – USP, São Paulo, Protocol number 2285/2007; Brazil). Signed informed consent was obtained from all participants. All patients (n = 29) had at least two positive serology tests for Chagas' disease, as determined by ELISA, immunofluorescence or hemagglutination techniques. All patients underwent a detailed clinical evaluation, 12-lead rest electrocardiogram (EKG), chest X-ray and a 2D-echocardiogram. Twenty one patients had not received etiologic treatment and 8 had received full treatment with benznidazole (5 mg/kg/day) for roughly 60 days. According to their clinical and laboratory characteristics (Table 1), the chagasic patients were divided in 3 groups: **Group 1 (n = 10):** Patients not treated with benznidazole and not showing signs of or only having mild cardiomyopathy, **Group 2 (n = 11):** Patients not treated with benznidazole but with moderate/severe cardiomyopathy, **Group 3 (n = 8):** Patients previously treated with benznidazole (cardiomyopathy-free or mild cardiomyopathy patients). Healthy Individuals from the same endemic areas were included in this study as controls, composing the **Group 4 (n = 10)**. All of them presented negative serologic tests for Chagas' disease and were matched by age and gender with the Chagas' disease patients.

**Table 1:** Demographic and clinical characteristics of chronic chagasic subjects included in this investigation.

Patient	Birth region	Gender	Age (years)	Clinical form	LVEF
1	SP	Male	47	Cardiomyopathy Free	54%
2	SP	Male	52	Cardiomyopathy Free	65%
3	MG	Male	59	Cardiomyopathy Free	73%
4	SP	Female	53	Mild Cardiomyopathy	68%
5	SP	Male	63	Mild Cardiomyopathy	65%
6	GO	Female	52	Mild Cardiomyopathy	71%
7	SP	Female	63	Mild Cardiomyopathy	54%
8	SP	Female	60	Mild Cardiomyopathy	50%
9	GO	Male	56	Mild Cardiomyopathy	49%
10	MG	Male	42	Mild Cardiomyopathy	57%
11	MG	Male	50	Moderate Cardiomyopathy	48%
12	MG	Female	57	Moderate Cardiomyopathy	47%
13	SP	Female	48	Moderate Cardiomyopathy	39%
14	GO	Male	57	Moderate Cardiomyopathy	40%
15	BA	Male	40	Moderate Cardiomyopathy	48%
16	SP	Female	66	Moderate Cardiomyopathy	49%
17	SP	Female	60	Severe Cardiomyopathy	34%
18	MG	Female	50	Severe Cardiomyopathy	15%
19	MG	Male	50	Severe Cardiomyopathy	32%
20	MG	Female	44	Severe Cardiomyopathy	24%
21	SP	Male	62	Severe Cardiomyopathy	35%
22*	GO	F	52	Bz 20 yrs Cardiomyopathy Free	67%
23*	GO	F	65	Bz 29 yrs Cardiomyopathy Free	61%
24 *	GO	M	76	Bz 35 yrs Cardiomyopathy Free	64%
25 *	GO	M	49	Bz 10 yrs Cardiomyopathy Free	-
26 *	GO	F	53	Bz 3 yrs Mild Cardiomyopathy	60%
27 *	GO	M	79	Bz 28 yrs Mild Cardiomyopathy	70%
28 *	GO	M	80	Bz 34 yrs Mild Cardiomyopathy	61%
29 *	GO	F	71	Bz 34 yrs Mild Cardiomyopathy	70%

*: Subjects 22, 23, 24, 25, 26, 27, 28 and 29 = with previous etiologic treatment (3 to 35 years ago); Bz = benznidazole.

Cardiomyopathy-free patients: asymptomatic, normal physical examination, normal ECG, normal chest X-rays and normal 2D-echocardiogram. LVEF>50%.

Patients with mild cardiomyopathy : positive symptoms or physical abnormalities, or abnormal ECG and/or chest X-rays and abnormal 2D-echocardiogram but with preserved global left ventricular function (LVEF>50%); moderate cardiomyopathy : impaired global LV function but EF still >35%; severe cardiomyopathy : LVEF≤35%.

### *T. cruzi* antigen

Protein lysate of *T. cruzi* (Y strain) obtained from LLMCK2 fibroblast cell line was used as the source of antigens. Briefly, the parasites were harvested, washed and submitted to 6 freeze/thaw cycles in liquid nitrogen and 37°C. The lysate was centrifuged at 12,000 g, the supernatant collected and the protein concentration determined.

### Isolation and culture of PBMC

Peripheral blood was harvested with heparin (50 U/mL) from healthy individuals and Chagas' disease patients. PBMC were isolated using Ficoll-Hypaque (Pharmacia Biotech) density gradient centrifugation, washed, counted, and used for CD4^+^CD25^+^ T cell isolation or cultured with specific antigen. PBMC (5×10^6^ cells/mL) were cultured for 48 h with *T. cruzi* antigen (10 µg/mL) and phytohaemagglutinin (PHA) (1 µg/mL) (Sigma-Aldrich, St. Louis) in 48 wells plates (final volume of 0.5 mL) and labeled with specific antibodies for phenotypic analysis in flow cytometer and determination of cytokine production in the supernatant of PBMC. As the concentration of IL-17 peaked at 48 h culture, we choose this time point for supernatant collection and cytokine assay.

### Surface markers (CTLA-4, CD103, GITR), Forkhead box P3 (Foxp3) and IL-17 detection

The cultured PBMC were washed in cold phosphate buffered saline (PBS) and samples of 5×10^5^ cells/tube incubated for 30 min at 4°C with PBS-5% rabbit normal serum to block unspecific bidding, followed by the addition of 0.5 µg of phycoerythrin (PE), allophycocyanin (APC) or fluorescein isothiocyanate (FITC)-labeled antibodies anti-CD3, anti-CD4, anti-CD25, anti-GITR, anti-CTLA-4 and anti-CD103 (all from BD-Pharmingen) for additional 30 minutes at 4°C in the dark. To detect the intracellular expression of Foxp3 the cells were fixed with cytofix/cytoperm solution (BD Biosciences) for 15 min at room temperature (RT), washed and stained with anti-Foxp3 or anti-IL17 peridinin chlorophyll protein (PERCP)-labeled, for 30 min at 4°C in the dark. Subsequently, the cells were washed twice and suspended in 100 µL of PBS-1% formaldehyde. In the assays involving intracellular detection of IL-17, the cells were incubated for additional 6 h in the presence of GolgiStop solution, according manufacturer's recommendations (BD Biosciences) and then treated as described above.

### Flow cytometry acquisition and analysis

Data acquisition was performed using a FACSCanto II (BD) and the multivariate data analysis performed with the FlowJo software (Treestar, USA), after collecting 50,000 events/sample. Distinct gating strategies were used to analyze the regulatory T cell and IL-17-producing CD4 T cell. Characterization of Treg started with gating the lymphocytes on FSC *versus* SSC dot plot. The T-lymphocyte subpopulations were further selected on FL1 ? anti-CD4 versus FL2 ? anti-CD25 dot plots. The percentage of cells expressing CTLA-4, CD-103, GITR and Foxp3 were analyzed in CD4 T cells, considering three different gates, according to the level of expression (or not) of CD25. The percentage of cells expressing intracellular IL-17 was analyzed within the gate of CD3^+^CD4^+^ population.

### Cytokine quantification (ELISA)

Cytokine production was assayed in supernatant culture of PBMC stimulated or not with *T. cruzi* antigen. ELISA sets were IL-10, IL-17, IFN-γ and TNF-α (R&D, Minneapolis, MN), and procedures were undertaken according to manufacturers' instructions. Optical densities were measured at 450 ηm. Results are expressed as picograms per milliliter.

### Co-cultures and CFSE proliferation assays

To verify the regulatory function of CD4^+^CD25^+^ T cells isolated from PBMC of moderate/severe cardiomyopathy or free/mild cardiomyopathy patients, they were cultured with PBMC (2×10^5^/well) from normal donors, at ratio 1∶5 and 1∶10, in 96-well U-bottom plates, in presence of PHA (1 µg/mL), at 37°C and 5% CO_2_. CFSE (Molecular Probes) was added at a final concentration of 1.25 µM. The solution was well mixed and incubated at RT for 5 min. An equal volume of serum was used to quench the reaction, after which, the cells were washed with PBS with 5% serum. On day 3 of culture, lymphocytes were collected, washed twice and suspended in 100 µL of PBS-1% formaldehyde. Data acquisition was performed using a FACSCanto II and the multivariate data analysis was performed in the FlowJo software. The data expressed as percentage of inhibition were calculated based on the PHA-induced proliferation of allogeneic T cells cultured without CD4^+^CD25^+^ T cells.

### Statistical analysis

Statistical analysis was performed using Mann-Whitney or Kruskal–Wallis tests, performed for the comparison of two or three variables between groups (INSTAT Software; GraphPad). The association between IFN-γ levels, regulatory T cell activity and left ventricular ejection fraction were tested by using the Spearman correlation (INSTAT Software; GraphPad). All values were considered significantly different at P<0.05.

## Results

### High frequency of CD4^+^IL-17^+^ T cell in PBMC from cardiomyopathy-free/mild patients

**Figure 1:**
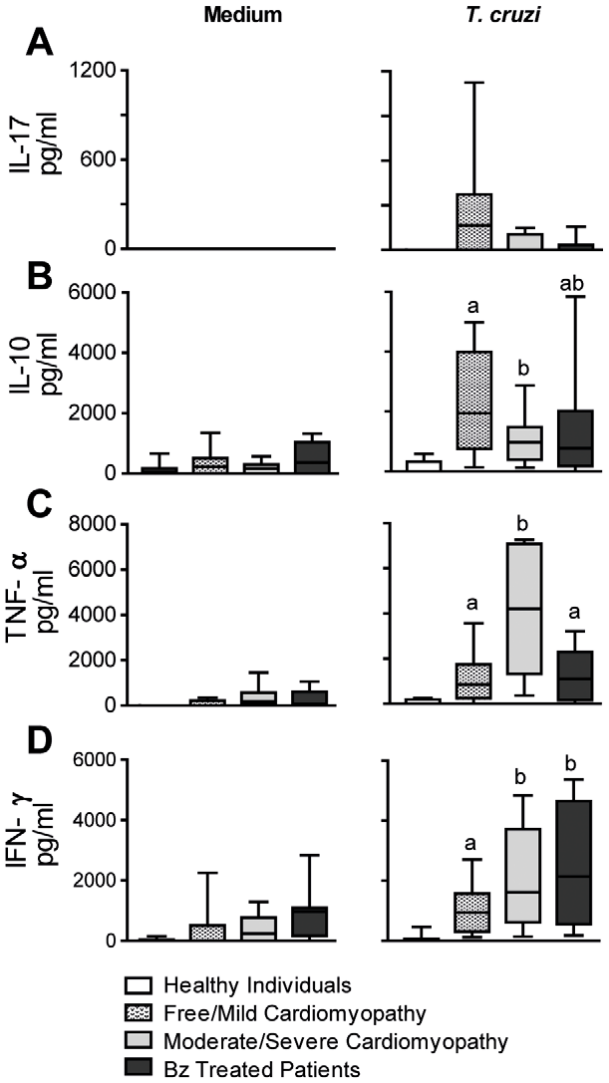
Higher IL-10 and lower TNF-α and IFN-γ secretion in free/mild *vs.* moderate/severe cardiomyopathy patients' PBMC. Levels of cytokines IL-17 (**A**), IL-10 (**B**), TNF-α (**C**) and IFN-γ (**D**) as examined by enzyme-linked immunosorbent assay in PBMC culture supernatants (5×10^6^ cells/mL in a 48 plate well) from patients, after 48 h of antigenic stimulation with trypomastigote antigen (100ηg/well) and independent of the stimuli (Medium). The Chagas' disease patients were grouped as: Group 1 (n = 10): Patients not treated with benznidazole and free/mild cardiomyopathy, group 2 (n = 11): Patients not treated with benznidazole but with moderate/severe cardiomyopathy, group 3 (n = 8): Patients previously treated with benznidazole free/mild cardiomyopathy. Healthy Individuals (n = 10) from the same endemic areas were included in this study as controls, composing the group 4, as described in Materials and Methods. The results are expressed in picograms per milliliter. Statistical differences are represented by letters: a and b, *P*<0.05 (Spearman).

**Figure 2:**
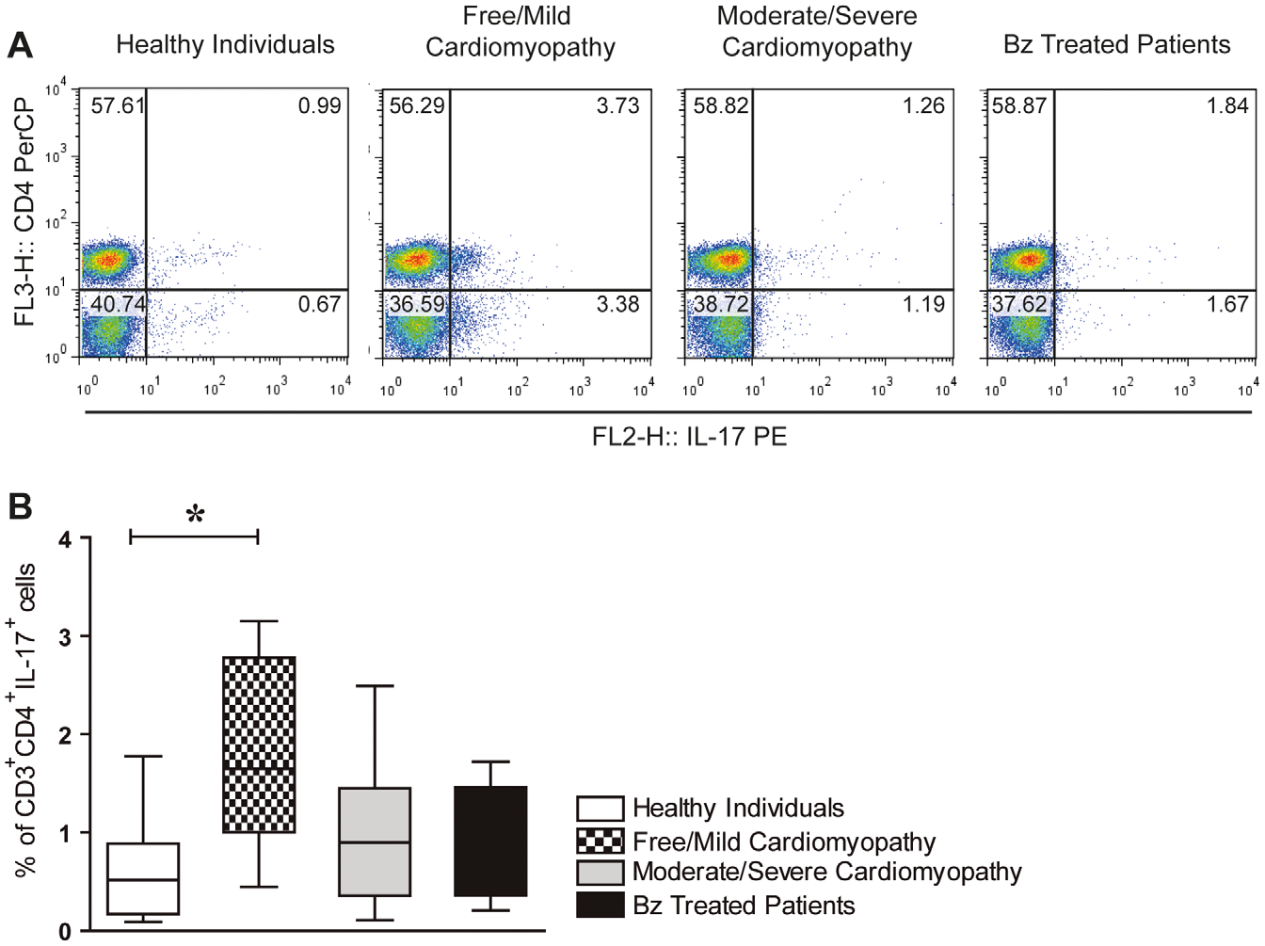
Increased frequency of CD4^+^IL-17^+^ Tcells in PBMC from free/mild cardiomyopathy patients. To examine the existence of Th17 lymphocytes in chronic Chagas' disease patients, PBMC (5×10^6^ cells/ml in a 48 plate well) from patients were cultured by 48 h with trypomastigote antigen (10 µg/mL) and the intracellular expression of IL-17 determined in CD3^+^CD4^+^ T cells by flow cytometry. PBMC from control and Chagas' disease patients in these analyses were gated on lymphocytes via their forward (FSC) and side scatter (SSC) properties, and CD3^+^CD4^+^IL-17^+^ were analyzed to determine the Th17 population. Representatives flow cytometry analysis of CD3^+^CD4^+^IL-17^+^ T cells gated lymphocytes from, Healthy individuals, Free/Mild cardiomyopathy patients, Moderate/Severe cardiomyopathy patients and Bz-treated patients are shown in **A**., while **B** shows the grouped analyses of all subjects in each group. Chagas' disease patients were grouped as: Group 1 (n = 10): Patients not treated with benznidazole and free/mild cardiomyopathy, group 2 (n = 11): Patients not treated with benznidazole but with moderate/severe cardiomyopathy, group 3 (n = 8): Patients previously treated with benznidazole free/mild cardiomyopathy. Healthy Individuals (n = 10) from the same endemic areas were included in this study as controls, composing the group 4, as described in Materials and Methods.

We first aimed to study the ability of cells from patients with different forms of the disease to produce IL-17, IL-10, IFN-γ and TNF-α after *T. cruzi* antigen stimuli. Similar levels of IL-17 were observed in all groups (**Figure 1A**). In contrast, cells from free/mild cardiomyopathy patients produced higher amounts of IL-10 than cells from moderate/severe cardiomyopathy patients group (**Figure 1B**). In addition, the response to *T. cruzi* antigen regarding the production of TNF-α and IFN-γ was higher in patients with moderate/severe cardiomyopathy (**Figure 1C, 1D**). The production of IL-17 by CD4^+^ T cells in PBMC from patients belonging to each experimental group, after being cultured with *T. cruzi* antigen obtained from trypomastigotes forms was also assessed using flow cytometry analysis. CD3^+^CD4^+^IL-17^+^ T cells from free/mild cardiomyopathy patients (3.73%) displayed increased frequency when compared to healthy individuals (0.99%). Conversely, moderate/severe cardiomyopathy patients (1.23%), Bz-treated patients (1.84%) and healthy individuals had similar frequency of these cells (representative dot plots are shown in Figure 2A). No significant differences were found in the intensities of IL-17 expression (MIF) in CD3^+^CD4^+^ T cells among the groups of Chagas' disease patients. When we analyzed the data obtained with the patients of all groups, we found that the percentage of CD4^+^T cells expressing IL-17 were expressively increased in the cardiomyopathy-free/mild group of patients (1.74±0.92) compared with all the other groups. The mean of the percentage of CD4^+^T cells expressing IL-17 in moderate/severe cardiomyopathy patients, Bz-treated patients and healthy individuals were 0.99±0.75, 0.90±0.58 and 0.67±0.57, respectively (**Figure 2B**). These findings were confirmed on confocal examination of PBMC.

### Patients with different clinical stages of Chagas' disease exhibit similar levels of CD4^+^CD25^high^ T cells

**Figure 3:**
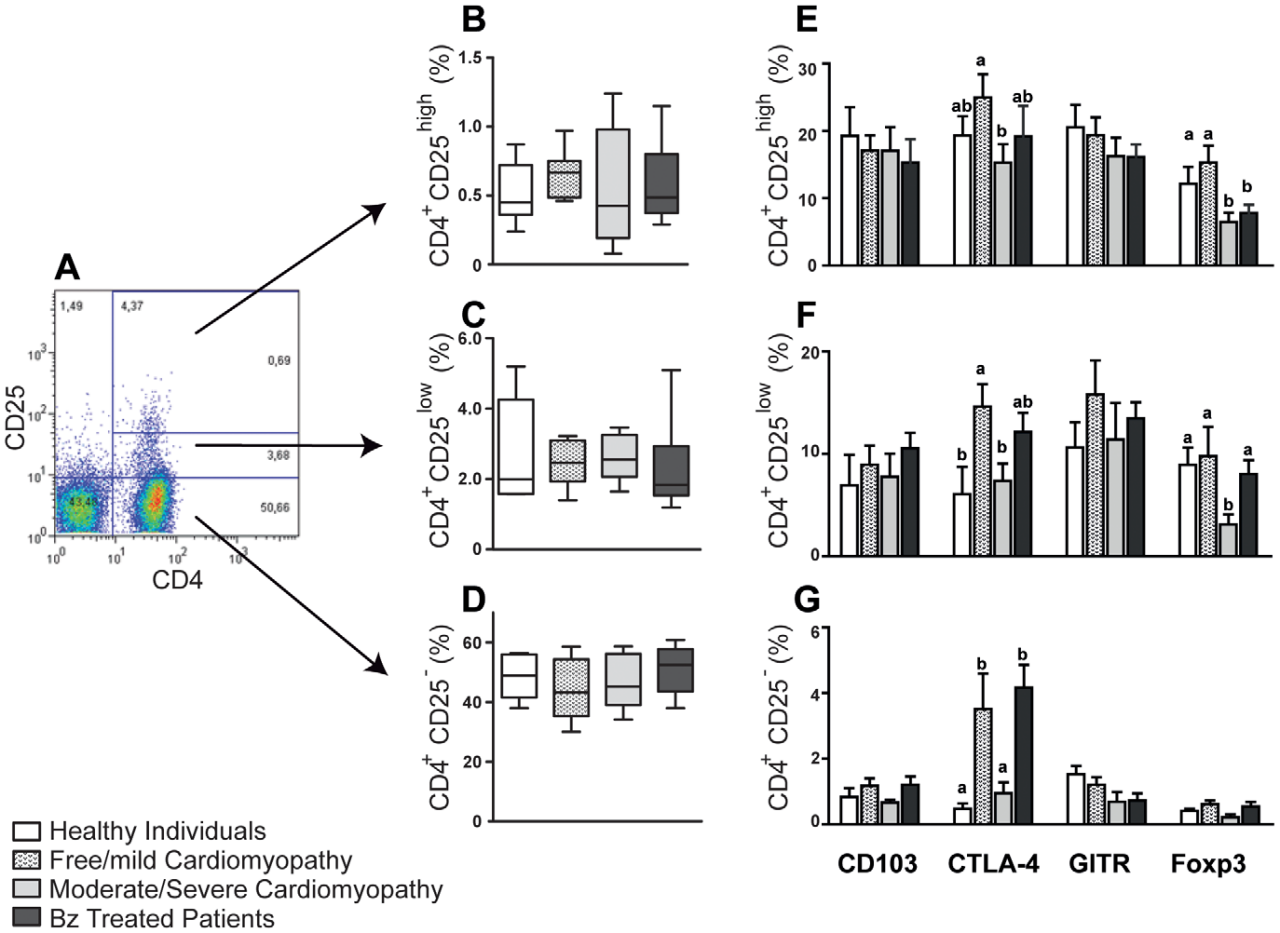
Characterization of CD4^+^CD25^+^ Treg in patients with different clinical manifestations of Chagas disease. Representative flow cytometry analysis of CD4^+^CD25^high^, CD4^+^CD25^+^Low, CD4^+^CD25^−^ gated lymphocytes is shown in **A**. PBMC from control and Chagas' disease patients in these analyses were gated on lymphocytes via their forward (FSC) and side scatter (SSC) properties, and CD4^+^CD25^high^ (**B**), CD4^+^CD25^+^Low (**C**), CD4^+^CD25^−^ (**D**) were performed to determine the regulatory T cell population. CD4^+^CD25^high^ (**E**), CD4^+^CD25^+^Low (**F**), CD4^+^CD25^−^ (**G**) cells were analyzed for their expression of membrane CTLA-4, GITR, CD103, and intracellular Foxp3. PBMC (5×10^6^ cells/mL in a 48 plate well) from Chagas' disease patients were cultured with trypomastigote antigen (10 µg/mL) after 48 h of antigenic stimulation the expression of surface markers (CD4, CD25, CD103, CTLA-4, GITR) and transcriptional factor (Foxp3) were determined. The results are expressed as means ± standard errors. **a** and **b** indicate statistical differences with P<0.05.

To characterize Treg population, CD4 versus CD25 dot plots were done and CD25^+^ lymphocytes classified in low and high or CD25^−^ T cells (as in Figure 3A). No significant differences in the frequencies of CD4^+^CD25^high^, CD4^+^CD25^low^ and CD4^+^CD25^−^ T cells were found among patients presenting different clinical forms of the disease as well as in controls (P = 0.118 comparing healthy vs. free/mild cardiomyopathy; P = 0.893, healthy vs. moderate/severe; P = 0.438, healthy vs. treated; P = 0.109, free/mild vs. moderate/severe cardiomyopathy; P = 0.247, free/mild vs. treated; P = 0.494, moderate/severe cardiomyopathy vs. treated) (**Figure 3B, C and D**). These results suggest that assessing the percentage of CD4^+^CD25^+^ could not be a reliable immunological approach to predict the different clinical forms of Chagas' disease.

### Increased frequencies of CD4^+^CD25^+^ T cells expressing CTLA-4 and Foxp3 in free/mild cardiomyopathy patients

**Figure 4:**
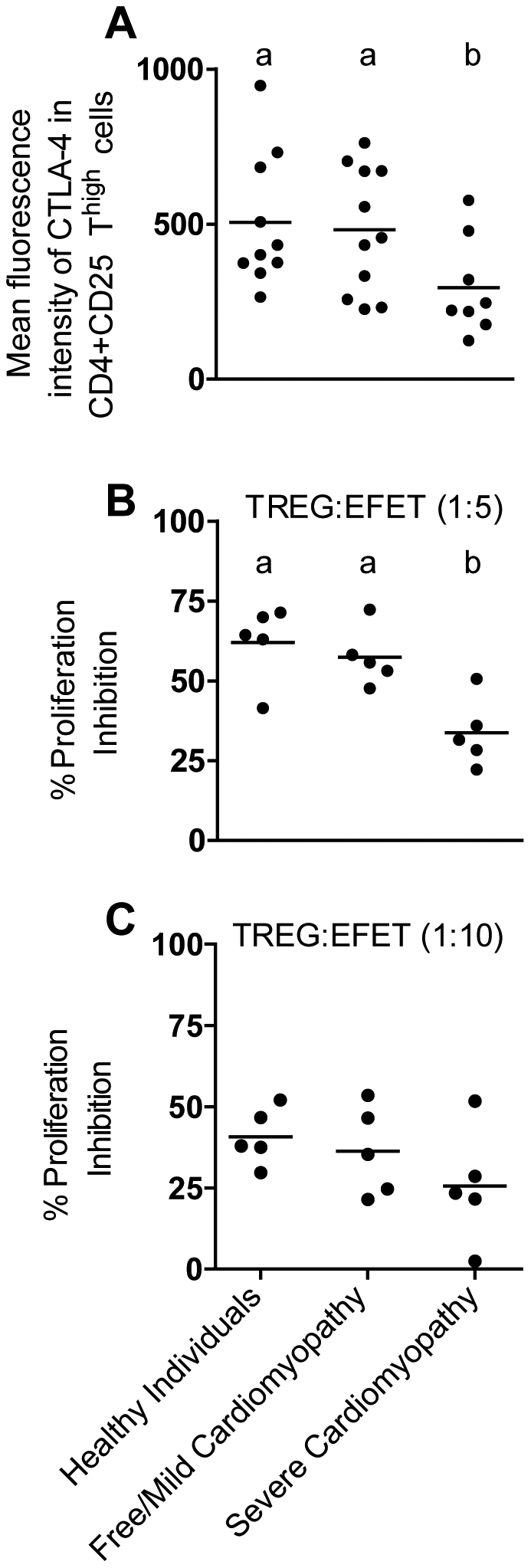
Patients with severe Chagas' disease cardiomyopathy exhibit deficient suppressor activity of Treg. PBMC (5×10^6^ cells/mL in a 48 plate well) from Chagas' disease patients were cultured with trypomastigote antigen (10 µg/mL) after 48 h of antigenic stimulation the mean intensity of fluorescence of CTLA-4 in CD4^+^CD25^high^ T cells (**A**) were performed. Free/Mild cardiomyopathy patients displayed high levels of CTLA-4 in CD4^+^CD25^high^ T cells (free/mild cardiomyopathy *vs.* moderate/severe P = 0.0352). For functional characterization of CD4^+^CD25^+^ regulatory T cells in Chagas' disease patients magnetic bead-sorted CD4^+^CD25^+^ T cells purified from PBMC from free/mild cardiomyopathy patients (*n* = 5), severe cardiomyopathy (*n* = 5), and healthy individuals (*n* = 5), were tested for their ability to suppress the proliferation of allogeneic PBMC. The CD4^+^CD25^+^ T cells were harvested and suppressor activity determined as % of proliferation inhibition in culture from PBMC/CD4^+^CD25^+^ T cells 1∶5 (**B**) and 1∶10 (**C**) proportion. Allogeneic PBMCs (2×10^5^ cells/well in a 96 plate well) CFSE stained were cultured during 72 h with medium only, CD4^+^CD25^+^ (2×10^4^ and 4×10^4^ cells/well, ratio of 1∶5), PHA (10 ηg/well), PHA plus CD4^+^CD25^+^ (ratio of 1∶10 and 1∶5) from Chagas' disease patients (free/mild cardiomyopathy patients and severe cardiomyopathy patients) or healthy controls. **a** and **b** indicate statistical differences with P<0.05 (healthy *vs.* free/mild cardiomyopathy P = 0.547; healthy patients *vs.* moderate/severe P = 0.0159; free/mild cardiomyopathy *vs.* moderate/severe P = 0.0189).

We next determined the frequency of cell that co-express CD103, GITR, CTLA-4, and Foxp3 on CD4^+^ T cell expressing high, low or absence of CD25. Free/mild cardiomyopathy patients presented higher frequency of CD4^+^CD25^high^ T cells expressing Foxp3 (P = 0.033) and CTLA-4 (P = 0.042) than moderate/severe cardiomyopathy patients (**Figure 3E**). High percentage of CD4^+^CD25^+^Low T cells expressing Foxp3 (P = 0.016) and CTLA-4 (P = 0.046) were also observed in free/mild cardiomyopathy patients compared with moderate severe cardiomyopathy patients (**Figure 3F**). Moreover, severe/moderate cardiomyopathy patients showed lower frequency of CD4^+^CD25^−^ T cells expressing CTLA-4 (P = 0.035) than free/mild cardiomyopathy patients, and Bz treated Chagas' disease patients (**Figure 3G**). The mean intensity of fluorescence (MIF) of CTLA-4, CD103, GITR and Foxp3 was similar in all groups studied. Interestingly, the expression of CTLA-4, but not CD103, GITR and Foxp3, in CD4^+^CD25*^high^* T cells was decreased in moderate/severe cardiomyopathy compared with free/mild cardiomyopathy patients and health individuals (**Figure 4A**). These data show that CTLA-4 expression and frequency of CTL-4^+^ T cells correlates with less severe cardiac disease. Moreover, it may indicate that treatment with benznidazol, with the consequent parasite elimination, may have important implications in the cardiac disease progression.

### CD4^+^CD25^+^ T cells from patients with severe cardiomyopathy exhibit deficient suppressive activity

We next aimed to study if the reduced frequency of CD4^+^CD25^+^ T cell expressing CTLA-4 and Foxp3 that we found in severe cardiomyopathy patients correlated with deficient regulatory activities. CD4^+^CD25^+^ T cells from healthy individuals, free/mild cardiomyopathy patients and severe cardiomyopathy patients were sorted, and suppressive activity was evaluated *in vitro* through co-culture assay with allogeneic T cells stimulated with PHA. The purity of CD4^+^CD25^+^ T cells isolated from free/mild cardiomyopathy patients and severe cardiomyopathy patients were about 99%. Interestingly, the inhibitory activity of CD4^+^CD25^+^ T cells from healthy individuals (62.95±5.37) (P = 0.0159) and free/mild cardiomyopathy patients (57.40±9.18) (P = 0.0189) were significantly higher than that observed with CD4^+^CD25^+^ T cells from moderate/severe cardiomyopathy patients (33.76±4.67), when cultured at a ratio of 1∶5 Treg∶allogeneic T cell (**Figure 4B**). Of note, no differences were observed among the groups when the ratio of Treg∶effector was 1∶10, possible due to a dilution effect in suppressive activity of these cells (**Figure 4C**). The impairment in suppressive activity observed in CD4^+^CD25^+^ T cells from patients suffering from severe cardiomyopathy correlates with the observation of reduced amounts of CD4^+^CD25^+^ T cells expressing CTLA-4 and Foxp3 in this group of patients.

### Increased levels of IFN-γ and decreased frequency of CD4^+^IL-17^+^T and deficient regulatory T cell activity correlates with chronic morbidity from Chagas' disease

**Figure 5:**
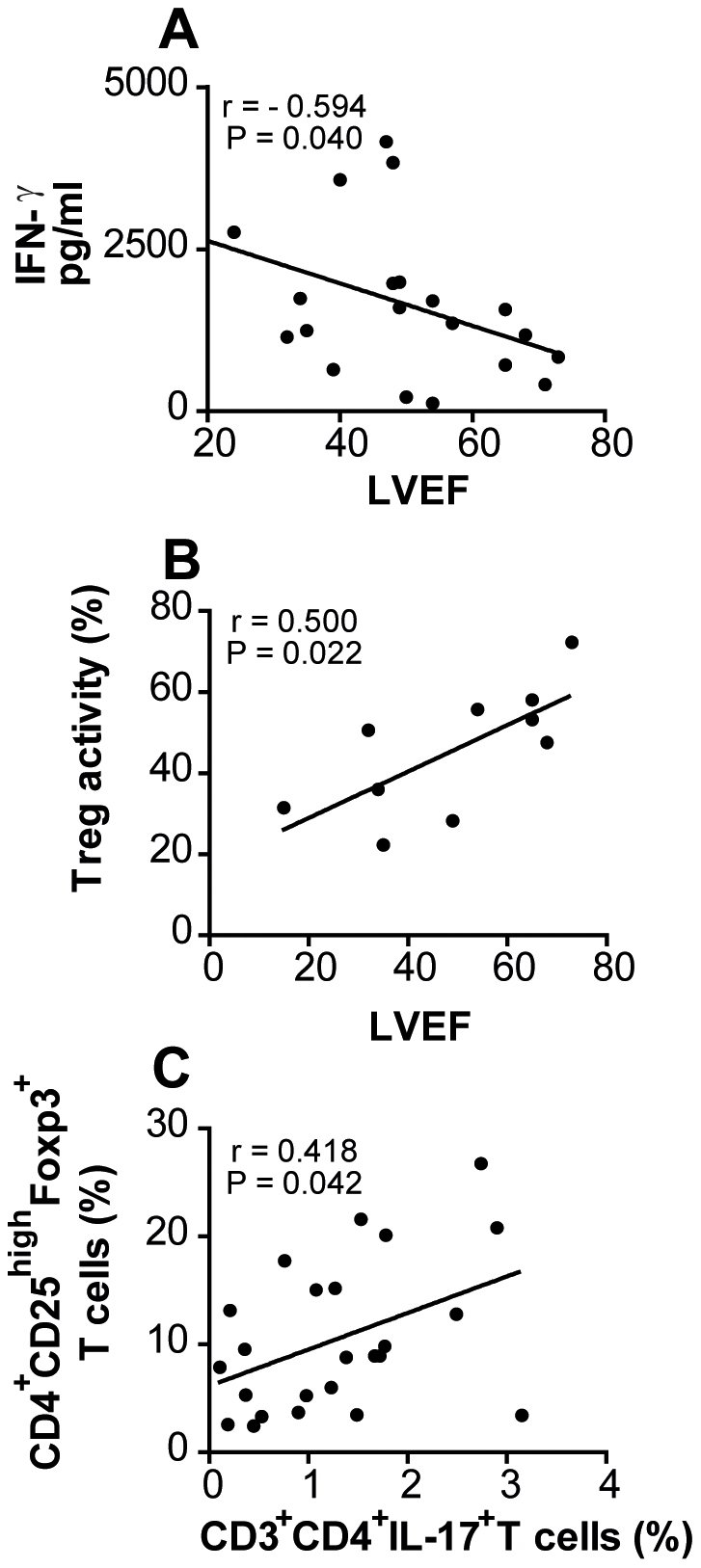
High Treg activity and reduced levels of IFN-γ correlate with normal LVEF. Correlation analyses were performed considering the values of LVEF and the levels IFN-γ in PBMC culture of patients (**A**) or *in vitro* suppressive activity of Treg (**B**). In **C** is showed the correlation between the frequency of CD4^+^CD25^high^Foxp3^+^ and CD3^+^CD4^+^IL-17^+^ T cells in PBMC from patients with the different clinical forms of Chagas' disease (free/mild cardiomyopathy patients, patients with moderate/severe cardiomyopathy and chagasic chronic patients treated with benznidazol after *in vitro* stimulation with *T. cruzi* antigens. The p values as long as the correlation coefficient are shown for each graph.

We next correlated LVEF with the levels of IFN-γ in the sera of all Chagas' disease patients and Treg suppressive activity obtained after allogeneic cultures (as described). Our results showed that IFN-γ levels during chronic Chagas' disease are inversely correlated to the LVEF (P = 0.040, r = −0.594) (Figure 5A). Accordingly, the levels of regulatory T cell activity are directly correlated with LVEF (P = 0.022, r = 0.500) (Figure 5B). We thus hypothesized that patients with chronic Chagas' disease undergoing cardiomyopathy produce increased levels of IL-17 and have a reduced frequency or suppressive activity of Treg compared with those patients with the indeterminate form of the disease. To our surprise, however, we found a positive correlation between frequency of CD4^+^IL-17^+^ T cell and CD4^+^CD25^+^HighFoxp3^+^ (P = 0.042, r = 0.418) (Figure 5C). In addition, no significant correlation was observed between TNF-α (P = 0.159, r = 0.133), IL-10 (P = 0.265, r = 0.066) production and LVEF.

## Discussion

In this investigation we first evaluated the production of IFN-γ, TNF-α, IL-10 and IL-17 in PBMC obtained from groups of Chagas' disease patients and in a group of benznidazol-treated individuals. The cultures of PBMC from patients with moderate/severe cardiomyopathy produced higher IFN-γ and TNF-α, and lower IL-10 levels than those observed in PBMC culture from free/mild cardiomyopathy patients, which is in accordance with previous reports by other researchers [1], [7], [9]. An imbalance in the production of cytokines IFN-γ and IL-10 was also observed in the present study, assaying these cytokines in the sera from chronic cardiac Chagas' disease patients: This imbalance has been implicated in the pathogenesis of Chagas heart disease [1], [7], [34]. Production of more IFN-γ and less IL-10 in cardiac patients supposedly results in efficient control of parasites replication but with more lesions to myocardium [5].

In addition, the analysis of IFN-γ production by ELISPOT of CD8 T cells from Chagas' disease patients showed that the frequency of IFN-γ producing-CD8 T cells is very low among those patients suffering the most severe form of the disease, and among individuals living in areas of active transmission of the disease, indicating that severe Chagas' cardiomyopathy could be related with the frequency of IFN-γ – producing T cells [31], [35]. On the other hand, one study comparing the levels of mRNA expression for the cytokines IL-5, IL-10, IL-13 and IFN-γ in PBMC from healthy individuals, and patients with cardiomyopathy or indeterminate forms of Chagas disease, found no differences among these groups [11]. Hence, there is not a consensus regarding the exact participation of classic Th1 cytokine profiles in the mechanisms that lead to the cardiac lesions during Chagas' disease.

It is therefore possible that other cytokine and cellular profiles participate in the immunological imbalance observed during Chagas' disease. One candidate is IL-17 which has effectively been involved in the control of parasites and in the induction of myocarditis in *T. cruzi* experimental infection [24]. In the present study PBMC from free/mild cardiomyopathy patients exhibited a higher expression of IL-17 in CD4^+^ T cells than that observed in PBMC from patients with severe/moderate cardiomyopathy and in cells from healthy individuals. Likewise, in the experimental model the inhibition of IL-17 resulted in enhanced production of IFN-γ and increased cardiac inflammation [24]. Moreover, impaired activation of immune-related cells that are critical for the killing of *T. cruzi* is observed in the absence of IL-17A gene [25]. Our data confirmed that PBMC from the group of moderate/severe cardiomyopathy patients produce more IFN-γ and TNF-α and less IL-10 than the cells obtained from the other groups. Cells from the same group of patients expressed more IL-17 when cultured with parasite antigens. In the same way, the infection with the trypanosomatid *Leishmania donovani*, the etiological agent of Kala Azar (KA), stimulates the differentiation into Th17 cells in PBMC obtained from healthy donors, leading to IL-17 and IFN-γ production [36].

As a result, IL-17 should be important in the control of cardiac inflammation by playing a negative feedback role on the production of IFN-γ and chemokines during *T. cruzi* infection in humans and mice, modulating the cardiac immune-mediated lesions found in Chagas' disease patients. Here we showed that the production of IL-17 is increased in patients without or with mild cardiac manifestations of the disease, which together with the results showing efficient suppressive activity of Treg in the same group of patients, suggest that IL-17 may be involved in the control of the immune response and, therefore, in the modulation of cardiac disease progression. As pointed before, IL-17 is also crucial for the control of parasite growth and host survival [24], [25]. These data are in agreement with that from a study on a cohort of subjects during a severe outbreak of the infection by the trypanosomatid *L. donovani*, in which the analysis of Th1, Th2, and Th17 cytokine responses by cultured PBMCs from revealed that IL-17 is associated with protection against severe KA [32].

The frequency of CD4^+^CD25^+^ regulatory T cells among patients with different clinical forms of Chagas disease was also examined in the present study. Surprisingly, all groups of patients showed a similar frequency of CD4^+^CD25^+^ T cell and CD4^+^CD25^high^ T cells. This is not in agreement with a previous report showing lower values of CD4^+^CD25^high^ T cells among school children with the indeterminate form of Chagas disease than that values observed in healthy children [17]. However, the same authors reported later in a study that patients with the indeterminate form of Chagas' disease exhibited a higher frequency of CD4^+^CD25^high^ T cell expressing Foxp3 and IL-10 as compared to those individuals with cardiomyopathy [37]. The last study was confirmed by a recent report showing that asymptomatic patients had increased amounts of Treg than those with cardiomyopathy [38]. Thus, it is possible to assume that a low frequency of regulatory T cell during early stages of Chagas' heart disease might be associated with the development of more serious chronic manifestations of Chagas' heart disease. As pointed out before, our study does not confirm these data probably due to our very well characterized groups of patients. All patients underwent a detailed clinical evaluation, 12-lead rest electrocardiogram (EKG), chest X-ray and a 2D-echocardiogram. However, in the experimental model of Chagas' disease the inhibition of Treg function with anti-GITR markedly increased the parasitemia, myocarditis and mortality compared with control mice [39].

As we did not detect differences in the percentages of Treg between the groups of patients in the present study, we investigated the suppressive activity of these cells. First, we assayed the expression of surface markers (CD103, CTLA-4 and GITR) as well as the transcriptional factor Foxp3. A higher expression of CTLA-4 and Foxp3 in the CD4^+^CD25^high^ T cells from free/mild cardiomyopathy patients was observed, when compared to moderate/severe cardiomyopathy patients. Moreover, the analysis of CD4^+^CD25^Low^ cells population demonstrated that free/mild cardiomyopathy patients and Bz treated patients have a higher occurrence of CTLA-4 than moderate/severe cardiomyopathy patients. The high amount of CD4^+^CD25^+^ T cells expressing CTLA-4 and Foxp3 (that activate the regulatory T cell machinery) in CD4^+^ CD25^+^ T cells of free/mild cardiomyopathy and Bz treated patients may be related to high suppressor activity of these cells. Furthermore, the greater number of CD4^+^CD25^−^ T cells of free/mild cardiomyopathy patients and Bz treated patients expressing CTLA-4 than cells from moderate/severe cardiomyopathy patients, probably contributes to the modulation of immune response in the heart. A higher incidence of T cells expressing CTLA-4 among CD4^+^CD25^−^ T cells from free/mild cardiomyopathy patients when compared to moderate/severe cardiomyopathy patients, also suggest a better negative control of the immune response, since CTLA-4 expression in CD25^−^ T cells is known to suppress the immune response [40].

The suppressive activity of Treg in PBMC from all groups of patients herein described was examined based in their capacity to suppress T cell proliferation. As we suspected, CD4^+^CD25^+^ T cells from Chagas' disease patients with severe cardiomyopathy presented reduced capacity to suppress T cell proliferation when compared to free/mild cardiomyopathy patients and healthy individuals. This phenomenon may be correlated by low frequency of CTLA-4 in the CD4^+^CD25^−^ T cells from cardiac patients. Nevertheless, it was previously reported that cardiomyopathy patients exhibit a higher percentage of CD4^+^CD25^high^ T cells expressing CTLA-4 [33]. The mechanism leading to reduced expression of Foxp3 and CTLA-4 and consequently, deficient suppressive activity of CD4^+^CD25^+^ T cells from patients with cardiomyopathy has not been elucidated. It is possible that a defective control of the immune response by Treg/Th17 may contribute to the pathogenesis of Chagas' heart disease, in a similar way as patients with other inflammatory and autoimmune diseases such as multiple sclerosis, systemic lupus erythematous, type 1 diabetes, psoriasis and rheumatoid arthritis have compromised functional activity of Treg [41], [42].

We also analyzed the levels of cytokines produced by PBMC from patients after *in vitro* stimulation with *T. cruzi* antigens, and we showed that IFN-γ production during chronic Chagas' disease is inversely correlated to LVEF, while normal regulatory T cell activity directly correlates with it. In addition, TNF-α production levels were lower in free/mild cardiomyopathy patients than in patients with moderate/severe cardiomyopathy. This finding is in agreement with a previous study reporting that patients with significant left ventricular (LV) dysfunction (LV ejection fraction ≤50%) showed higher levels of TNF-α, compared to Chagas' disease patients without LV dysfunction [43]. Moreover, studies in patients with dilated cardiomyopathy reported a significant increase of TNF-α among these individuals when compared with healthy controls, suggesting that the elevation of TNF-α could be an immune pathogenic mechanism in the progression to cardiomyopathy. Here we showed that the production of TNF-α (and not IFN-γ) tends to be lower among benznidazole-treated individuals. Although further research are required to explore the mechanisms by which benznidazole can induce these differential effects on cytokines production, these findings has been experimentally addressed before, and coincide with our current results. For example, it was shown that IFN-γ mediates the protective effect of benznidazole against *T. cruzi* infection [44], and slightly inhibits the synthesis of TNF-α in murine cells [45]. The levels of this cytokine may also constitute an important marker of ventricular dysfunction in chronic chagasic cardiomyopathy [46], [47].

One import result found in the present study was a positive correlation between IL-17 and Foxp3 expression in PBMC among Chagas' disease patients. Therefore, more IL-17 and Foxp3 expression is preferentially found in free/mild cardiomyopathy patients. Thus, the expanded Treg are better able to control the inflammatory response in presence of Th17. This data are in agreement with the previous demonstration that Th17 are preferentially differentiated in the presence of Treg [28] due the consumption of IL-2 by Treg [26], [27] Chronic autoimmune inflammation originates when this process is deregulated, and then therapeutic intervention becomes necessary to restore that balance between Th17 and Treg.

It is clear that genetic characteristics of both the host and the parasite are important in determining the outcome of the infection. Our data suggest that genetic aspects of the immune response involved in the functions of Treg, IL-17, and some related genes may deserve further investigation and may shed light on the comprehension of the immune pathogenesis of Chagas' disease.

In summary, our results show that CD4^+^CD25^+^ Treg from patients with severe cardiomyopathy display a deficient suppressive activity, leading to uncontrolled production of pro-inflammatory cytokines (TNF-α and IFN-γ) from leukocytes. Moreover, patients with less aggressive forms of the disease (cardiomyopathy free or mild cardiomyopathy individuals) produce higher levels of the cytokines IL-10 and IL-17. Reduced CD4^+^CD25^+^ regulatory T cell function and low levels of IL-17 also correlated with more advanced cardiomyopathy. We think that these findings may be helpful in the design of immunotherapeutic approaches for eventual primary, secondary and tertiary prevention of chronic Chagas' cardiomyopathy.
